# A giant infiltrating angiolipoma of the mediastinum: a case report

**DOI:** 10.1186/s13019-016-0560-6

**Published:** 2016-12-01

**Authors:** Peng Liu, Wen-Cheng Che, Huai-Jun Ji, Zhong-Min Jiang

**Affiliations:** 1 Taishan Medical College, Taian, China; 2 Shandong University School of Medicine, Jinan, China; 3 Weifang Medical College, Weifang, China; 4 Department of Thoracic Surgery, Shandong Qianfoshan Hospital, Jingshi Road No.16766 Lixia, Jinan, Shandong China

**Keywords:** Infiltrating angiolipoma, Mediastinum, Thoracotomy

## Abstract

**Background:**

Angiolipoma is a rare benign neoplasm composed of mature fatty tissue and multiple small abnormal blood vessels. Infiltrating mediastinal angiolipoma is an extremely rare tumor associated with delayed diagnosis.

**Case presentation:**

A 42-year-old woman was presented with 3-month history of mild chest tightness. Imaging of the chest showed a large mass with fat densities in the middle superior mediastinum. A presumptive diagnosis was a tumor of liposarcoma. The patient was scheduled for a thoracotomy. After the excision, the symptoms were relieved and histological study revealed that the tumor was an angiolipoma. The patient recovered very well and was discharged 7 days after the surgery. After 7 months of follow-up the patient was clinically well and asymptomatic.

**Conclusions:**

We described a giant infiltrating mediastinal angiolipoma and its removal, and discussed the tumor characteristics and prognosis. Although extremely rare, infiltrating angiolipoma should be considered in the differential diagnosis of mediastinum lesions. The prognosis after surgical management of our patient is favorable.

## Background

Mediastinal angiolipomas are extremely rare tumors within the thorax, and only several cases have been previously reported in the literature. The diagnosis and prognosis of angiolipomas are based entirely on the findings of histological evaluations. Angiolipoma has two histologic types: infiltrating and non-infiltrating [1]. We report a rare case of giant infiltrating mediastinal angiolipoma. To our knowledge, this is the first report of an infiltrating angiolipoma in mediastinum.

## Case presentation

**Fig. 1:**
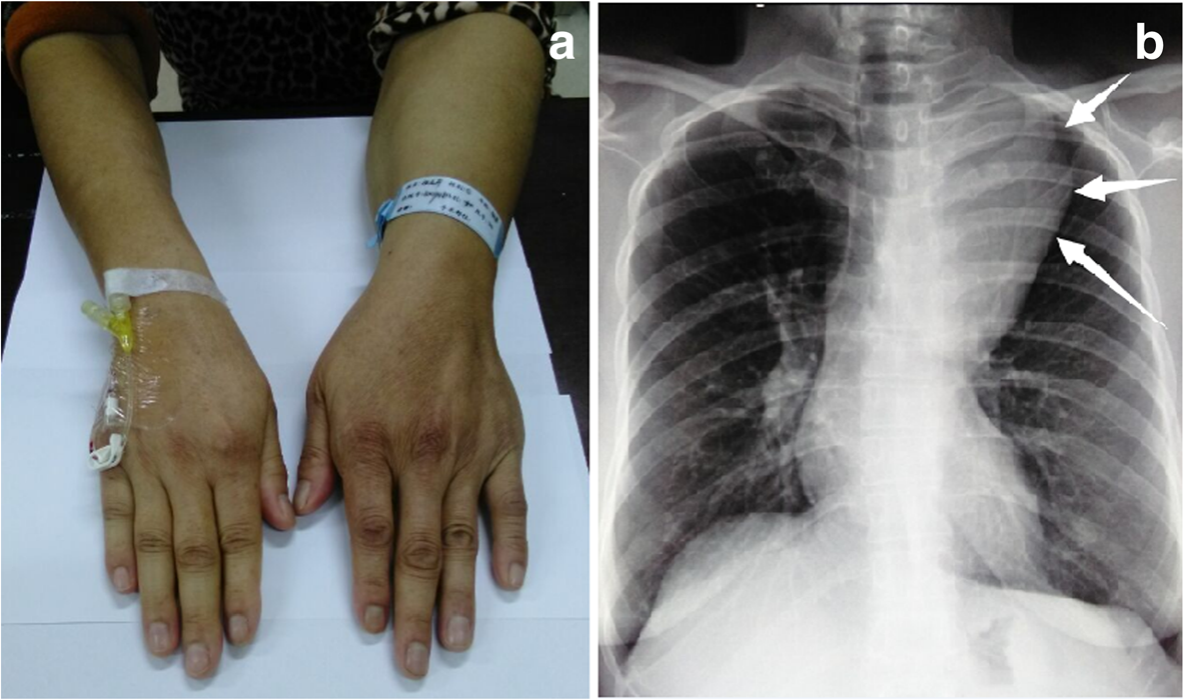
**a** The left arm muscles were thicker than the contralateral ones. **b** X-ray of the chest revealed a mass in the superior mediastinum

**Fig. 2:**
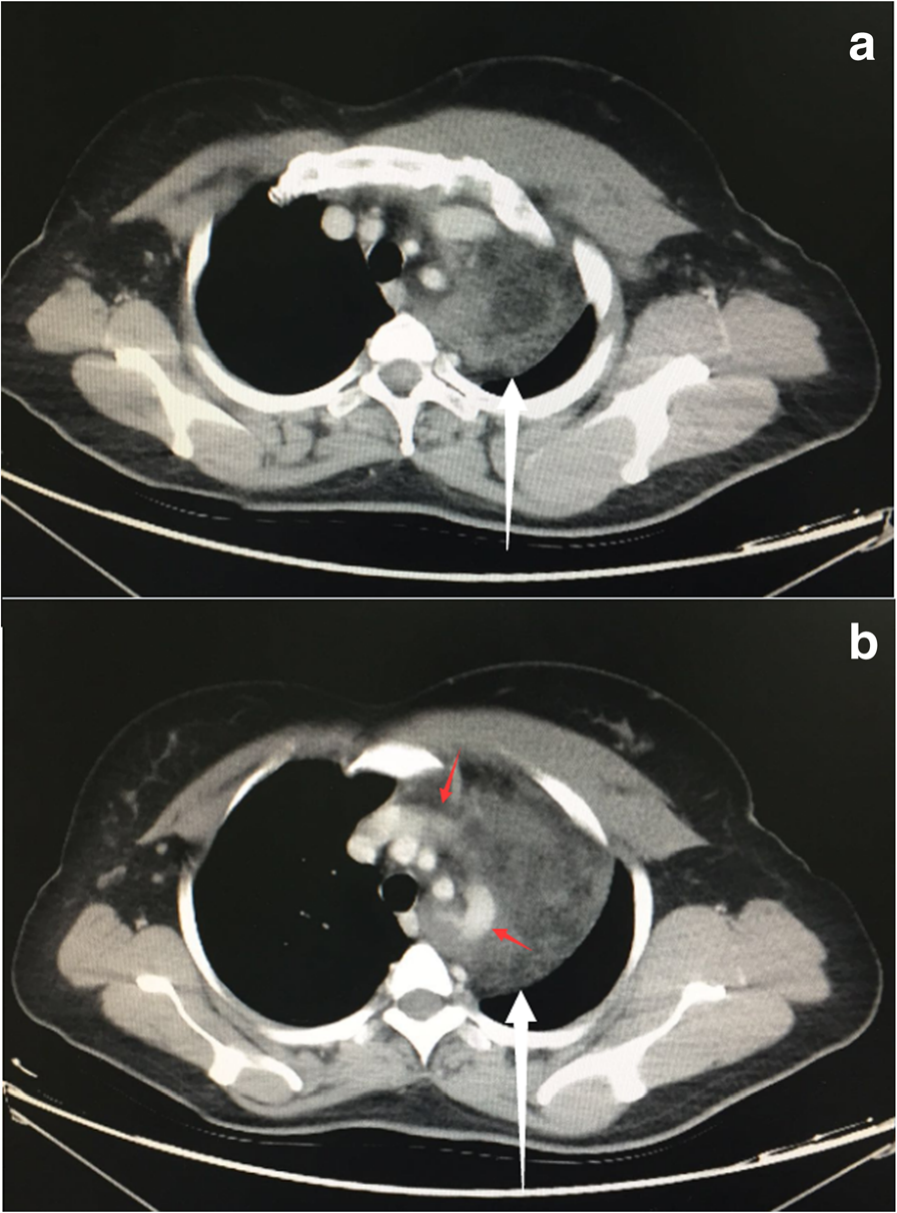
**a** CT scan of chest showed a 10*5.5 cm-sized mass (*white arrow*) with fat densities in the middle superior mediastinum. **b** The tumor was slightly heterogeneously enhanced and the adjacent blood vessel was surrounded by the lesion with obscure boundary(*red arrow*). The left chest and back muscles were thicker than the contralateral ones

A 42-year-old female presented with 3-month history of mild chest tightness. Her medical history was unremarkable. Physical examination found that her left arm muscles were thicker than the contralateral ones (Fig. 1a). Breath sounds on auscultation were diminished at the upper left lung. Plain X-ray of the chest revealed a mass in the superior mediastinum (Fig. 1b). Computed tomography (CT) of the chest showed a mass, measuring 10 cm × 5.5 cm in size with fat densities in the middle superior mediastinum (Fig. 2a). On the post contrast images the tumor was slightly heterogeneously enhanced and the adjacent blood vessel was surrounded by the lesion with obscure boundary (Fig. 2b). Laboratory tests were negative.

**Fig. 3:**
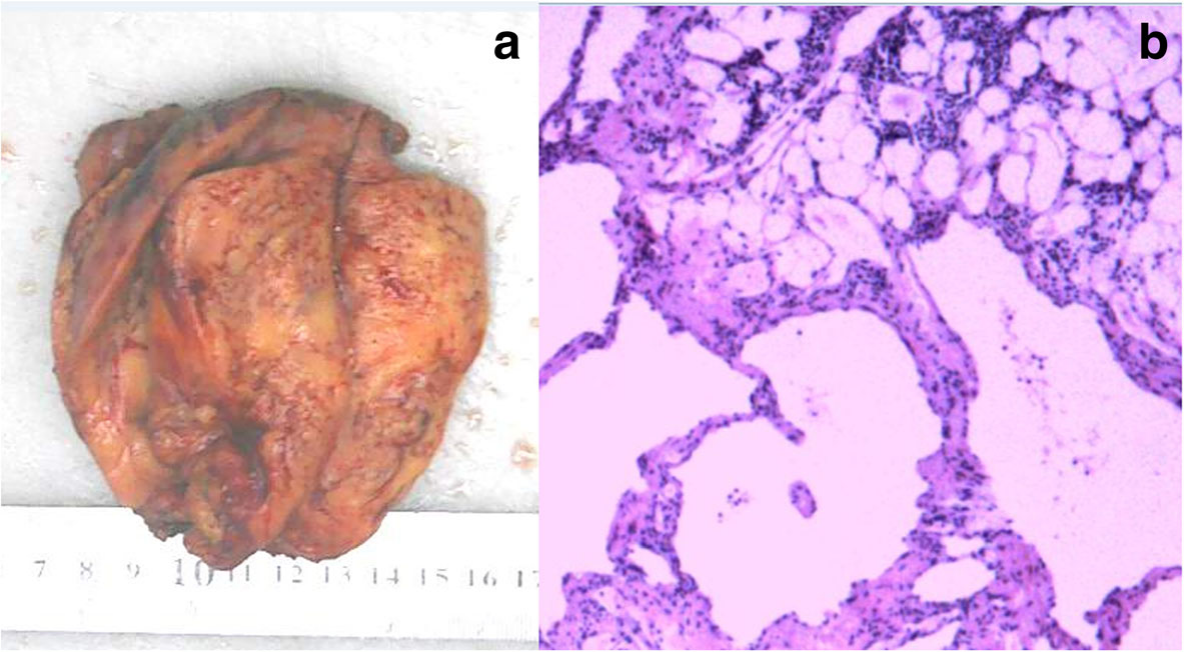
**a** Excised tumor, a 10*6*6 cm-sized hard mass with a *gray-yellowish* in color and a sense of oily. **b** H and E stained section showing features of angiolipoma

This large infiltrating mass was considered a tumor of liposarcoma. Taking into account the provisions of China’s health insurance and the practice of local surgeons, a biopsy during operation should be done to determine the pathological type. The patient was scheduled for a thoracotomy. During the operation, a large mass, invaded the left subclavian artery and brachiocephalic vein, was found in the anterior mediastinum. Since it was very difficult to dissect the mass completely from the mediastinal structures, especially the left subclavian artery, an incomplete resection was performed. Macroscopically, it was revealed a hard mass of 10 cm × 6 cm × 6 cm in size with a gray-yellowish in color and a sense of oily (Fig. 3a). Pathologic evaluation demonstrated mature adipose tissue with many hyperplastic blood vessels, being consistent with the typical findings of angiolipoma (Fig. 3b). The patient recovered very well and was discharged 7 days after the surgery. Considering the incomplete resection, we recommened that the patient undergo a strategic radiation therapy after 1 month of the surgery. After 7 months of follow-up the patient was clinically well and asymptomatic.

## Discussion

Angiolipoma is a variant of lipoma exhibiting proliferating capillaries admixed with mature adipocytes. Angiolipoma was first defined by Bowen in 1912 [2] and differentiated from lipomas histopathologically by Howard in 1960 [3]. Angiolipoma has two histologic types: infiltrating and non-infiltrating [1]. Infiltrating angiolipomas are characterized by a non-capsulated tumor and extend into surrounding tissues. This case was a giant infiltrating angiolipoma of 10-cm diameter located in the mediastinum and invaded the adjacent tissues. Non-infiltrating angiolipomas are encapsulated. Angiolipomas usually present as painful or tender subcutaneous masses in young adults. Infiltrating angiolipomas can also lead to muscular pain and neural deficits [1]. The thickened left arm, in this case, may be the results of muscular compensatory hypertrophy caused by a poor blood supply resulting from the long-term compression of the left subclavian artery by the large mediastinal mass.

Mediastinum lesions have a wide range of pathologic features but a similar imaging appearance. The most common tumors found in the anterior mediastinal compartment are of thymic, lymphatic, or germ cell origin. More rarely, neoplasms and other masses originating from vascular or mesenchymal tissues may also be found. Taking into account the patient’s age, the location of the mass and its radiological appearance, the differential diagnosis in this case could have included teratoma, lipoma, and liposarcoma.

Teratoma is one of the most common tumors found in the mediastinum, and mostly is located in the anterior mediastinal compartment. Mediastinal teratomas are often asymptomatic. When such symptoms as chest pain, cough, dyspnea, or symptoms related to recurrent pneumonitis are present, they are usually attributed to mechanical effects. Mediastinal teratomas are occasionally discovered incidentally on chest radiograph. The enlarging teratoma can also result in local compression symptoms that make the differential diagnosis more challenging. But, in this case, bhCG, AFP and other laboratory tests were normal, which did not support the diagnosis of teratoma.

Lipomas are common benign mesenchymal tumors. More than half of lipomas encountered by clinicians are subcutaneous in location. But they may develop in virtually all organs throughout the body. Numerous case reports document the presence of lipomas in other, rare locations, with these tumors having been found virtually everywhere in the body. In rare instances, lipomatous involvement of the structural components of the mediastinum, including the airways and pleura, has also been reported. In this case, the large tumor in the mediastinum caused a chest tightness, and impinged on the adjacent blood vessels, including the left subclavian artery and brachiocephalic vein.

Liposarcomas are malignant tumors that arise from adipocytes. They may recur locally and may metastasize to distant organs. Fatty tumors with poor capsule of the mediastinum should be considered to be potential liposarcomas until proven otherwise. In addition, liposarcomas most commonly arise in the posterior mediastinum, as opposed to the anterior predilection of both lipomas and teratomas [2].

There is no evidence that angiolipomas undergo malignant transformation due to lack of atypia, pleomorphism, or mitotic figures in angiomatous or adipose tissues. Treatment of angiolipomas is complete surgical excision for both infiltrating and non-infiltrating types. In some cases, however, it is difficult for infiltrating type of angiolipomas to be removed completely by surgical approach. In this case, the recurrence rate is high and postoperative radiotherapy may be needed [1].

A search for cases of mediastinal angiolipoma revealed that only a limited number documented cases have been reported. Kline in the year 1990 first reported a case of non-infiltrating angiolipoma of the mediastinum. A CT scan showed a large and soft-tissue components near the esophagus [2]. After his initial report, Ja-Young Choi in 2000 also reported a case of posterior mediastinal angiolipoma extending into spinal canal and showing both fat and angiomatous features on CT scan [4]. Luis Gorospe in 2015 described a lipid-poor angiolipoma within the posterior mediastinum in a patient with chest pain [5]. Occurrence of infiltrating angiolipomas of mediastinum is extremely rare.

## Conclusions

We report a case of extra-large infiltrating angiolipoma of the mediastinum. Although extremely rare, infiltrating angiolipoma should be considered in the differential diagnosis of mediastinum lesions. In addition, the large infiltrating mass in mediastinum may invade the surrounding structures and the procedure of dissection and removal may be very difficult. Clinicians should be aware of this.

## Abbreviation

AFP: Alpha-fetoprotein
